# The Missense Variant in the Signal Peptide of *α-GLA* Gene, c.13 A/G, Promotes Endoplasmic Reticular Stress and the Related Pathway’s Activation

**DOI:** 10.3390/genes15070947

**Published:** 2024-07-19

**Authors:** Sabrina Bossio, Ida Daniela Perrotta, Danilo Lofaro, Daniele La Russa, Vittoria Rago, Renzo Bonofiglio, Rosita Greco, Michele Andreucci, Antonio Aversa, Antonella La Russa, Anna Perri

**Affiliations:** 1 Department of Experimental and Clinical Medicine, University “Magna Graecia”, 88100 Catanzaro, Italy; sabrina.bossio@unicz.it (S.B.); aversa@unicz.it (A.A.); 2 Department of Biology, Ecology and Earth Sciences, Centre for Microscopy and Microanalysis (CM2), University of Calabria, 87036 Rende, Italy; ida.perrotta@unical.it; 3 e-Health Lab, Department of Mechanical, Energy, Management Engineering, University of Calabria, 87036 Rende, Italy; danilo.lofaro@unical.it; 4 Department of Pharmacy, Health and Nutritional Sciences, University of Calabria, 87036 Rende, Italy; daniele.larussa@unical.it (D.L.R.); vittoria.rago@unical.it (V.R.); 5 Kidney and Transplantation Research Center, Annunziata Hospital, 87100 Cosenza, Italy; rbonofi@gmail.com; 6 Nephrology, Dialysis, and Kidney Transplant Unit, Annunziata Hospital, 87100 Cosenza, Italy; r.greco@aocs.it; 7 Department of Health Sciences, Magna Graecia University, 88100 Catanzaro, Italy; andreucci@unicz.it (M.A.); a.larussa@unicz.it (A.L.R.)

**Keywords:** Anderson–Fabry disease, αGLA genetic variants, signal peptide, endoplasmic reticulum stress

## Abstract

Anderson–Fabry disease (AFD) is an X-linked multisystemic disorder with a heterogeneous phenotype, resulting from deficiency of the lysosomal enzyme α-galactosidase A (α-Gal A) and leading to globotriaosylceramide systemic accumulation. Lysosomal storage is not the unique player in organ failure and different mechanisms could drive tissue damage, including endoplasmic reticulum (ER) stress and its related signaling pathway’s activation. We identified a new missense variant in the signal peptide of *α-GLA* gene, c.13 A/G, in a 55-year-old woman affected by chronic kidney disease, acroparesthesia, hypohidrosis, and deafness and exhibiting normal values of lysoGb3 and αGLA activity. The functional study of the new variant performed by its overexpression in HEK293T cells showed an increased protein expression of a key ER stress marker, GRP78, the pro-apoptotic BAX, the negative regulator of cell cycle p21, the pro-inflammatory cytokine, IL1β, together with pNFkB, and the pro-fibrotic marker, N-cadherin. Transmission electron microscopy showed signs of ER injury and intra-lysosomal inclusions. The proband’s PBMC exhibited higher expression of TGFβ 1 and pNFkB compared to control. Our findings suggest that the new variant, although it did not affect enzymatic activity, could cause cellular damage by affecting ER homeostasis and promoting apoptosis, inflammation, and fibrosis. Further studies are needed to demonstrate the variant’s contribution to cellular and tissue damage.

## 1. Introduction

Anderson–Fabry disease (AFD) is a rare X-linked lysosomal enzymopathy due to pathogenic mutations in the galactosidase α gene (*αGLA*), leading to a partial or total deficit of the lysosomal enzyme GLA. The functional enzymatic deficiency results in an intra-lysosomal accumulation of complex lipids (Gb3/LysoGb3), leading to progressive tissue damage and multiple organ failure, with predominant involvement of the heart, kidneys, and nervous system [1]. The pathogenesis of AFD has been fully described, but the molecular mechanisms underlining organ dysfunction have not yet been completely elucidated, and growing experimental and clinical evidence suggests that the tissue damage is triggered by the activation of different intricate pathways, such as immune inflammatory response, fibrosis, apoptosis, and autophagy [2,3]. 

More than 1000 Fabry disease-associated mutations have been identified, and about 70% of them are missense mutations [4]. Many mutations are “private” as they only occur in one family, but the high phenotypic variability, even between individuals with the same pathogenic variant, makes it very difficult to establish genotype–phenotype correlations. Therefore, it strengthened the hypothesis that, beyond the mutation, many other factors can influence the phenotype’s severity and the onset age of the disease in both males and females. Furthermore, affected individuals may be carriers of gene variants of uncertain significance (GVUS), whose pathogenetic role in the phenotype expression of the disease has not been established yet [5].

Most of the secretory proteins, such as α-GLA are synthesized as precursors, that is, pre-proteins with an additional N-terminal short sequence of 15 to >50 amino acids, named a signal peptide (SP) that serves to target the pre-protein to the endoplasmic reticulum (ER). After or during the translocation to the ER, the SP is removed by the signal peptidase complex [6,7]. Some studies reported that SPs can affect both the stability and folding of several proteins [8,9,10,11]. 

ER retention of misfolded proteins can lead to stress and activate the unfolded protein response (URP) that, in turn, triggers downstream pathways to reduce protein synthesis and increase ER-associated folding and degradation. Glucose-regulated protein 78 (GRP78) is the key leader of UPR in the ER, forcing the unfolded proteins to refold or degrade using cellular degradation mechanisms [12]. When the cell is exposed to accumulated unfolded proteins in the ER, GRP78 is released from the UPR sensors and moves to the cell surface, playing an essential role in intracellular signaling, proliferation, migration, invasion, apoptosis, sterile inflammation, and immunity [13,14,15]. Therefore, when the homeostasis of the ER cannot be restored, those pathways activated by ER stress can promote cellular damage. 

In this study, we investigated the intracellular effect of a not yet described missense variant in the signal peptide of *α-GLA* protein, c.13 A/G (p.Asn5Asp), which we identified in a 55-year-old woman with suspicious ADF.

## 2. Materials and Methods

### 2.1. Genetic Analysis

After acquiring written informed consent, genomic DNA was extracted from peripheral blood leukocytes by using the Wizard Genomic DNA Purification kit (Promega, Madison, WI, USA), following the manufacturer’s instructions. At a later stage, DNA was quantified by spectrophotometric reading absorbance at 260 nm with Eppendorf BioSpectrometers (Eppendorf, Milan, Italy). Mutational analysis was performed by exon PCR of the *α-GLA* gene using the primers used in the literature [16]. PCR products were purified using a Centri Spin-40 (Applied Biosystems, Thermo Fisher, Waltham, MA, USA) and sequenced using a Genetic Analyzer 3700 (Applied Biosystems, Thermo Fisher, Waltham, MA, USA); resulting sequences were evaluated with the SequencherTM software (version 5.4).

### 2.2. Cell Culture

HEK293T cells were grown in Dulbecco’s Modified Eagle’s medium (DMEM) with high glucose (Sigma-Aldrich, St. Louis, MO, USA) supplemented with 10% FBS, 1% L-glutamine, and 1 mg/mL penicillin/streptomycin (Sigma Aldrich, Milano, Italy).

### 2.3. Plasmid Amplification and Purification

The wild-type (WT) construct (20ACG5NC_Gene_A_pMA-T) and the construct carrying the variant (20ACG50C_Gene_G_pMA-T) were created by Invitrogen (Life Technologies, Darmstadt, Germany). Specifically, the synthetic gene GLA_gene_A and gene GLA_G were assembled from synthetic oligonucleotides and/or PCR products. Each fragment was inserted into the vector backbone pMA-T. The plasmid DNA was purified from transformed bacteria and the concentration was determined by UV spectroscopy. The final constructs were verified by sequencing at Life-Technologies’s Laboratory (Thermo Fisher, Waltham, MA, USA). The sequence identity within the insertion sites was 100%. Plasmids were transformed into DH5α competent cells and amplified in LB medium containing specific antibiotics overnight using a 37 °C shaker with speed at 225 rpm. Then, the bacteria were harvested, and plasmids were extracted and purified using a Qiagen MaxiPrep plasmid extraction kit (Hilden, Germany) according to the procedure provided by the manufacturer.

### 2.4. Transient Transfection

The plasmid WT (Gene A) and plasmid mutant (Gene G) were transiently overexpressed in HEK293T cells, after reaching approximately 70–90% confluence. The plasmids’ transfection into cells was performed using Lipofectamine 2000 (Invitrogen, Waltham, MA, USA), according to the manufacturer’s instructions. Briefly, 4 µgr of plasmids was mixed with 10 µL of Lipofectamine 2000 in 250 µL of Opti MEM reduced serum medium (Thermo Fisher, Waltham, MA, USA). After 20 min incubation at room temperature, the mixture was added to the HEK293T cells. After 24 h of transfection, the cells were harvested and used for mRNA and protein extraction, and TEM.

### 2.5. mRNA Extraction and Reverse Transcription–Polymerase Chain Reaction (RT-PCR)

For the preparation of RNA samples, after 24 h of transient transfection, cultured cells were rinsed with cold PBS and were solubilized in 1 mL of Trizol (Thermofisher, Waltham, MA, USA). The RNA samples were obtained by phenol/chloroform extraction. The RNA was precipitated with isopropanol and washed with 75% ethanol and dissolved in RNase-free water. The RNA was quantified by nanodrop and RT-PCR was performed according to the manufacturer’s instructions; 2 μg of total RNA was reverse transcribed in a final volume of 20 µL using the High Capacity cDNA Archive Kit (Applied Biosystems, Applera Italia, Monza, Milano, Italy). The cDNAs were amplified by PCR: 1 µL of first-strand cDNA, 1 mol/L each primer [16], 0.5 mmol/L dNTP, and Taq DNA polymerase (2 U per tube) (Promega Corp, Madison, WI, USA) in a final volume of 25 µL. A negative control containing water instead of first-strand cDNA was used. Cells transfected with empty vectors were used as control.

### 2.6. Protein Extraction and Western Blotting Analysis

For the preparation of cell lysates, after 24 h of transient transfection, cultured cells were rinsed with cold PBS and solubilized in an RIPA buffer (Cell Signaling Technology, Danvers, MA, USA), plus phenylmethanesulfonyl fluoride (PMSF, Sigma-Aldrich, St. Louis, MO, USA) at 1 mM concentration. The cell lysates were quantified spectrophotometrically using the Bio-Rad Bradford Assay (Bio-Rad Laboratories, Hercules, CA, USA). All of the samples were loaded on a 10% SDS–polyacrylamide gel, transferred to a nitrocellulose membrane, and probed with antibodies directed against GRP78, BAX, p21, pNFkB, IL1β, N-Cadherin, and TGFβ1 (Santa Cruz Biotechnology, Santa Cruz, CA, USA). As the internal control, all the membranes were probed with anty-glyceraldehyde-3-phosphate dehydrogenase, GAPDH antibody (Santa Cruz Biotechnology, Santa Cruz, CA, USA). The antigen–antibody complex was detected through incubation of the membranes for 1 h at room temperature with peroxidase-coupled goat anti-mouse, anti-rabbit, or anti-goat IgG and revealed using the enhanced chemiluminescence system (Clarity Western ECL Substrate, Biorad, Hercules, CA, USA). The blots were then exposed to film (Santa Cruz Biotechnology, Santa Cruz, CA, USA). The intensity of bands representing the relevant proteins was measured using ImageJ densitometry scanning software (Version 1.54j). 

### 2.7. Transmission Electron Microscopy (TEM)

Immediately after sample collection, cell pellets were gently washed with PBS and fixed in 3% glutaraldehyde solution in 0.1 M phosphate buffer (pH 7.4) for 2 h at 4 °C. After osmium tetroxide post-fixation and buffer washes, samples were dehydrated through acetone graded series (30%, 50%, 70%, 80%, 90%, and 100%) for 15 min twice per grade and then progressively embedded in acetone/resin with a final embedment in pure resin (Araldite–Fluka) (Sigma-Aldrich St. Louis, MO, USA) overnight at room temperature. Afterward, the samples were transferred to a fresh resin mixture in embedding capsules and polymerized in an oven at 60 °C for about 72 h. Ultrathin sections were cut with a diamond knife, mounted on copper grids (G300 Cu), and imaged using a JEM 1400-Plus electron microscope (Jeol S.p.A., Milan, Italy) operating at 80 kV. Cells exhibiting ER injury were quantified manually from electron micrographs at 8000 to 15,000× magnification according to their phenotype. Only fully longitudinally sectioned cells were counted. As previously reported, we calculated the percentage of cells with ER abnormalities based on 100 cells per condition [17].

### 2.8. Peripheral Blood Mononuclear Cell (PBMC) Isolation

PBMCs were isolated by density separation over a Ficoll-PaqueTM (GE Healthcare, Uppsala, Sweden) gradient (460 g for 30 min) and were washed three times with PBS pH 7.4/1 mM EDTA (Sigma, Milan, Italy). Total protein extraction from isolated PBMCs was performed as reported above.

### 2.9. Statistical Analysis

All experiments were performed in at least triplicates and repeated in three independent experiments. Optical densities were measured using the ImageJ software (Version 1.54j) and their results are presented as a ratio between Gene G/Gene A vs. control. All results are presented as mean ± SD of data from three combined experiments. Data were analyzed by Student’s *t*-test using GraphPad Prism 8.3.0 (GraphPad Software, Inc., San Diego, CA, USA). *p* < 0.05 was considered statistically significant and the figures were created using R (R version 4.2.1, RStudio version 2021.09.0).

## 3. Results

### 3.1. Clinical and Biochemical Features of the Proband

A 55-year-old Caucasian woman affected by chronic kidney disease was admitted to the Department of Nephrology of Cosenza Hospital. She suffered from acroparesthesia triggered by physical stress and exogenous heat since childhood, gastrointestinal disturbance, hypohidrosis, and deafness. She has a family history of end-stage renal disease of unknown origin. The routine biochemistry showed impaired renal function (serum creatinine 1.83 mg/dL; blood urea: 55 mg/dL; estimated glomerular filtration rate (eGFR): 48.8 mL/min) and proteinuria (300 mg/24 h). Autoimmune disorders were excluded. A transthoracic echocardiogram showed left ventricular hypertrophy (Ejection Fraction: 48%). The patient was submitted to genetic screening for ADF disease. The plasma concentration of lysoGb3 and the α-GLA activity were both in the normal range (1.09 nMol/L, reference value: 0.08–1.13 nMol/L; 8.0 nmol/mL/h; reference range: >3 nmol/mL/h, respectively—performed by IRIB CNR, Palermo, Italy). The genetic analysis performed on all family members showed that only the proband’s sisters had the variant, in the absence of clinical signs of disease. Males did not carry the variant. These data suggest that the variant was transmitted through the paternal line. The proband’s father died at the age of 66 because of acute myocardial infarction. He was affected by deafness. No further clinical information was available. The only child of the proband, a 32-year-old man, did not have the variant.

### 3.2. In Vitro Transfection of the Genetic Variant c.13 A/G, Codifying the Signal Peptide

**Figure 1:**
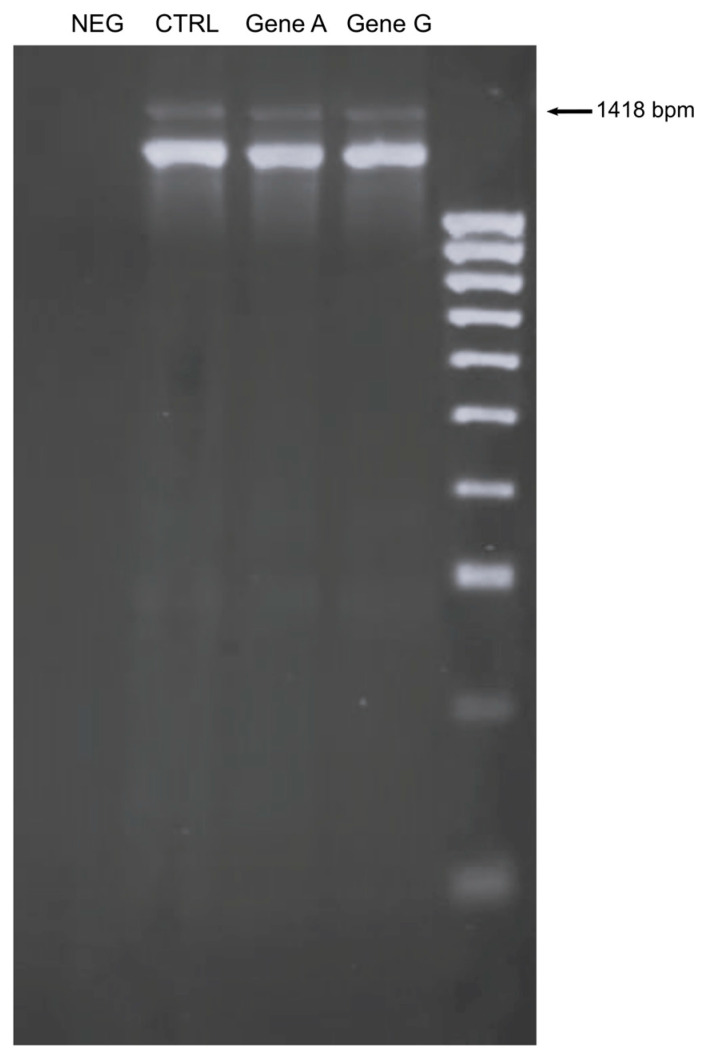
mRNA expression of αGLA in HEK293T transfected with Gene A and Gene G. Negative control (NEG), cells transfected with empty vector (CTRL).

DNA sequencing identified a new genetic variant in exon 1 of the *α-GLA* gene, c.13 A/G, which caused an asparagine-to-aspartic acid substitution (p.Asn5Asp) and codified the signal peptide. This variant has not been previously reported in the Human Gene Mutation Database or other mutation databases of FD. In addition, to the best of our knowledge, the variant has not been found in healthy subjects. The in vitro characterization of the genetic variant was conducted using Human Embryonic Kidney 293T Cells. The cells were used for the transfection of the full-length cDNA sequence encoding the wild-type human α-Gal A (20ACG5NC_Gene_A_pMA-T) and the new variant c.13 A/G (20ACG50C_Gene_G_pMA-T). The transfection confirmation was performed by GLA expression using PCR analysis (Figure 1). 

### 3.3. The Genetic Variant c.13 A/G Promotes ER Injury and Intra-Lysosomal Accumulation

**Figure 2:**
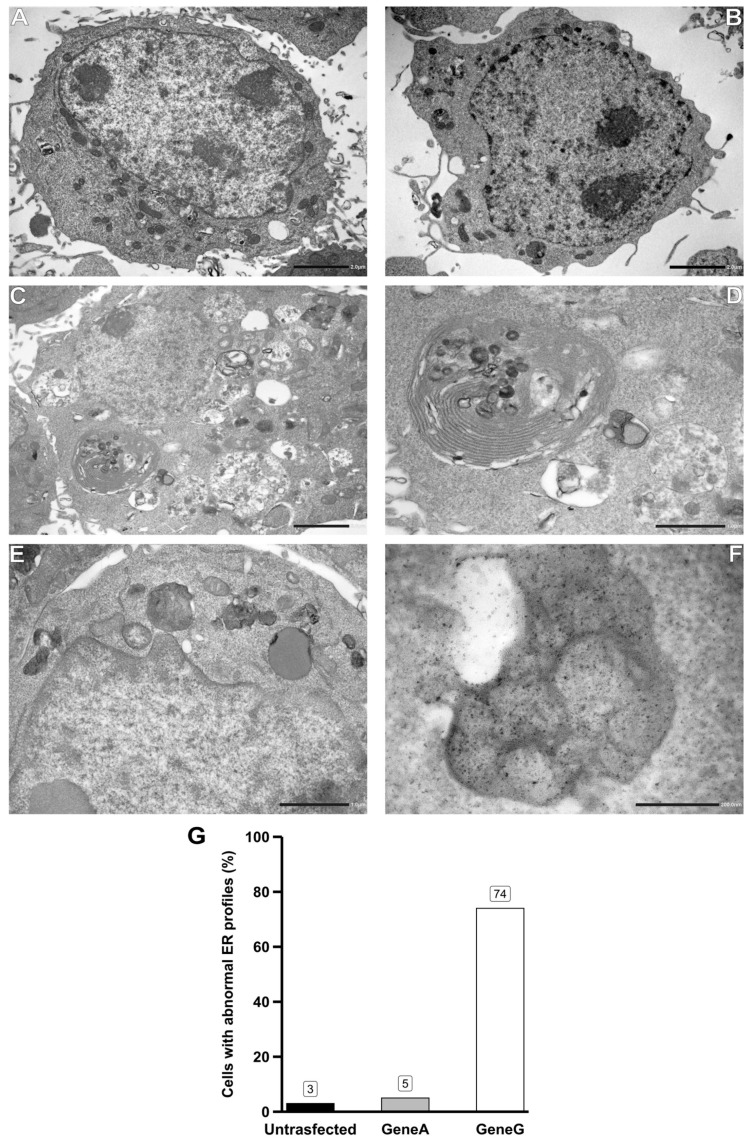
Electron micrographs showing HEK293T cells untransfected and transfected with Gene A and Gene G. Untransfected cell (**A**); cell transfected with Gene A (**B**): in both cells, mitochondria display regular shapes with parallel cristae regularly distributed within the matrix. The ER exhibits a tubular appearance. The nucleus maintains a uniform, spheroid shape with a well-defined nuclear membrane and contains multiple nucleoli. (**C**–**F**) Cell transfected with Gene G. (**C**,**D**) Cell contains large aggregates of altered ER membranes. The ER aggregates are frequently found in multilayered concentric whorls that enclose portions of the cytoplasm. (**E**) Increased number of lysosomes that often exhibit an abnormal morphology and contain storage material with granular and fingerprint profiles. (**F**) Accumulated autophagosomes. (**G**) The percentage of cells with ER abnormalities was calculated based on 100 cells per condition.

On transmission electron microscopy, control cells showed good ultrastructural preservation with well-preserved cell compartments and well-defined membranes. The cytoplasm contained a well-developed Golgi system, mitochondria, and abundant rough and smooth ER that consisted of an elaborate network of flat cisternae (or tubules) and lysosomes that appeared as moderately large bodies with round/oval shapes and a homogenous internal structure of high electron density (Figure 2A,B). When analyzing the cells transfected with Gene G, we noticed a marked reshaping of the ER that formed multiple concentric membrane structures or whorls, which are signs of ER injury (Figure 2C,D). Furthermore, to strengthen our findings, in our TME images, we determined the percentage of cells exhibiting ER injury. We observed that most cells untreated or transfected with Gene A showed regular arrays of ER membranes, whereas 74% of cells transfected with Gene G displayed altered ER appearance (Figure 2G). In addition, the lysosomes contained numerous lamellar inclusions with regular periodicity as well as fingerprint profiles which were absent in the control counterpart and only rarely observed in the cells transfected with the Gene A plasmid (Figure 2E,F). No morphological defects in other organelles, such as Golgi and mitochondria, were observed. The cell’s ultrastructure was free of visible artifacts in all cases examined.

### 3.4. The Genetic Variant c.13 A/G Triggers Pro-Apoptotic, Pro-Inflammatory, and Pro-Fibrotic Intracellular Signals by Increasing GRP78 Expression

**Figure 3:**
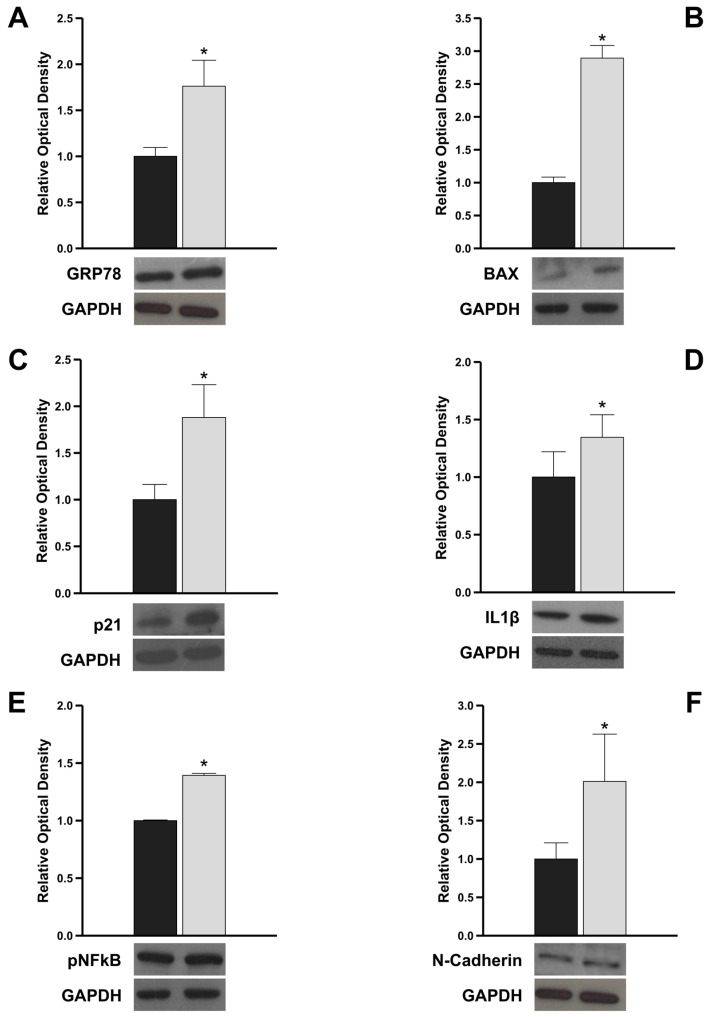
Protein expression levels of markers of ER stress, apoptosis, inflammation, and fibrosis in HEK293T transfected with Gene A and Gene G. Immunoblotting showing GRP78 (**A**), BAX (**B**), p21 (**C**), IL1β (**D**), pNFkB (**E**), and N-Cadherin (**F**) in HEK293T transfected with Gene A and Gene G. GAPDH was used as a loading control. The bars represent the mean ± SD of three separate experiments, in which the band intensities were evaluated as the optical density and are represented as fold change for Gene G vs. Gena A normalized for the loading control. * *p* < 0.05.

To investigate whether the structural features observed by TEM in cells transfected with Gene G were accompanied by an increase in ER stress markers, we evaluated the expression level of GRP78, using protein extracts obtained from cells transfected with Gene A and Gene G. It is well known that in standard conditions, GRP78 is bound in its inactive form to the UPR transmembrane stress sensors and that, upon stress conditions, such as the accumulation of unfolded proteins, GRP78 increases and is released from the UPR sensors [13]. Interestingly, we observed a significant upregulation of GRP78 in Gene G cells compared to that observed in Gene A cells (Figure 3A), suggesting that the variant could promote ER stress and the activation of the URP. Furthermore, in agreement with the evidence reported in the literature demonstrating that during prolonged ER stress, the URP complex can trigger apoptotic signaling [14,15], in Gene G cells, we observed a significant upregulation of the pro-apoptotic protein BAX and p21 that is a major regulator of cell cycle progression and apoptosis (Figure 3B). In addition, to explore whether the ER stress response induces sterile inflammation, we evaluated the protein content of a key pro-inflammatory cytokine, IL-1β, and the transcriptional factor NFkB, notoriously involved in the transcription of several pro-inflammatory cytokines. Western blot results revealed an increased expression of both IL-1β (Figure 3C) and pNFkB (Figure 3D) in Gene G cells compared to Gene A cells. Finally, to verify whether the pro-inflammatory state ER stress-induced could promote epithelial-to-mesenchymal-transition, we evaluated the expression level of the mesenchymal marker, N-Cadherin. Interestingly, we observed a significant upregulation of N-Cadherin in Gene G cells versus Gene A cells (Figure 3E), highlighting that the intracellular deleterious effect of the genetic variant could also result in the acquisition of a mesenchymal phenotype, leading to fibrosis. 

Overall, these results strongly suggest that the new variant activating URP could trigger pro-apoptotic, pro-inflammatory, and pro-fibrotic signals, leading to cellular injury.

### 3.5. Proband’s PBMCs Exhibit Pro-Inflammatory and Pro-Fibrotic Profile

**Figure 4:**
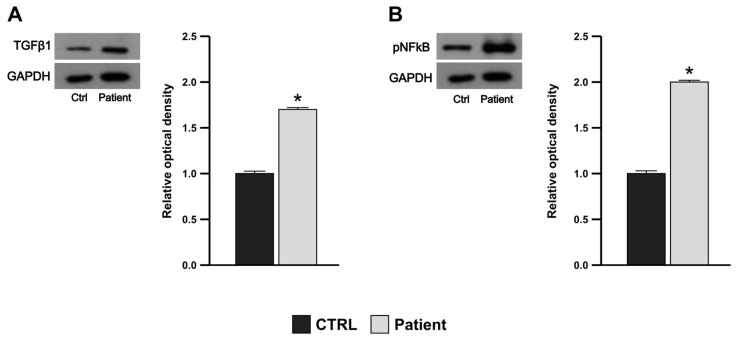
Increased TGFβ1 and pNFkB protein expression in patient’s PBMCs. Immunoblotting showing TGFβ1 (**A**) and pNFkB (**B**) protein expression of PBMCs from a healthy subject (Ctrl) and the patient. GAPDH was used as a loading control. The bars represent the mean ± SD of three separate experiments, in which the band intensities were evaluated as the optical density and are represented as fold change for patient vs. control (CTRL) normalized for the loading control. * *p* < 0.05.

To investigate the presence of pro-inflammatory and pro-fibrotic states in the PBMCs of the proband, we evaluated the protein expression levels of the profibrogenic cytokine transforming growth factor-β, TGFβ-1, the key leader in the transdifferentiation toward myofibroblasts of several types of cells, and of pNFkB. In line with our in vitro results, the Western blot analysis performed on total proteins extracted from PBMCs of the proband showed a strong and significant upregulation of both TGFβ-1 and pNFkB versus a healthy control, suggesting that the genetic variant could contribute to the pro-inflammatory and pro-fibrotic profile observed in PBMCs (Figure 4A,B). 

## 4. Discussion

The findings emerging from our in vitro study suggest that the new genetic variant identified in exon 1 of the *α-GLA* gene codifying the signal peptide of GLA, c.13 A/G, could promote ER stress and increase the cellular expression of some markers of apoptosis, inflammation, and fibrosis, highlighting that the activation of ER stress-related detrimental pathways could have a role in the cellular damage observed in AFD, in addition to GB3 intra-lysosomal accumulation.

Nascent polypeptides contain cleavable N-terminal signal sequences, named SPs, that mediate protein targeting and translocation into the ER. An important role of SPs is to favor the protein translocation in a selective and attenuated manner during ER stress to reduce the protein folding burden in the ER. Furthermore, some authors reported that SPs can regulate the timing of cleavage by the signal peptidase complex, enhancing protein maturation, and that SPs, in addition to mediating the direct interaction with the membrane for protein insertion to or across the membrane, are also involved in the folding of precursor proteins [8]. Earlier studies conducted on Escherichia coli precursor maltose binding protein demonstrated that SPs slow the protein’s folding and its translocation-competent conformation [9,18]. We used an in vitro methodological approach to investigate the intracellular impact of the newly identified variant. The TEM clearly showed that a large percentage (74%) of cells transfected with the plasmid carrying the variant exhibited a reshaping of the ER that formed multiple concentric membrane structures, which are signs of ER injury. Furthermore, we observed that the lysosomes contained numerous lamellar inclusions and fingerprint profiles absent in Gene A cells. These findings suggest that the variant could promote ER injury and, interestingly, intra-lysosomal inclusions that could not be LysoGb3 but other intermediate forms of reaction representing the so-called “isoforms” or analogs of Gb3 or LysoGb3, as reported by Tuttolomondo and colleagues [2]. However, additional experimental studies are needed to establish the significance of these inclusions. 

Our hypothesis that the presence of the variant could be associated with ER stress has been strengthened by the observation of a significant upregulation of GRP78 protein. It is well known that to counteract the detrimental effects of ER stress, cells activate a complex cellular response, URP, mediated by three ER transmembrane receptors that, in resting cells, are maintained in an inactive state through their association with the ER chaperone, GRP78. Upon stress conditions, GRP78 dissociates from the three receptors triggering the URP, aiming to reduce the accumulation of unfolded proteins and restore normal ER functioning [13,19]. However, when the homeostasis of the ER cannot be restored because of the injury’s persistence, the URP-mediated intracellular signaling switches from pro-survival to pro-apoptotic and pro-inflammatory, leading to cellular damage [14,15]. The molecular mechanisms underlying the switch from the protective URP signaling to the pro-apoptotic pathway activation upon prolonged ER stress have been extensively elucidated, highlighting that the phosphorylation of BCL2 family members, of which BAX is a part, by the downstream molecule JNK, is crucial to set the death machinery in motion [14]. Our results are in line with this evidence, as, interestingly, in cells transfected with the c.13 A/G variant, we detected an increased expression of the pro-apoptotic protein BAX and the negative cell cycle regulator, p21, compared to cells transfected with a wild type. Furthermore, different studies demonstrated that ER stress-induced URP signaling activation is associated with the production of many pro-inflammatory molecules, and that the cross-talk between inflammation and URP pathway activation could influence the pathogenesis and/or progression of diseases concerning cells of the immune system or metabolism [15]. According to this consolidated evidence, we observed that cells carrying the variant exhibited a pro-inflammatory state compared with control since we detected a significant upregulation of the pro-inflammatory IL-1β, as well as of the transcription factor NFkB, which is involved in the transcription of several pro-inflammatory cytokines, including IL-1β [20]. It is well known that the inflammatory profile can favor the acquisition of mesenchymal phenotype by cells, contributing to fibrotic changes that severely impair normal tissue architecture and function [21]. Interestingly, in Gene G cells, we observed a significant upregulation of N-Cadherin, a well-known marker of ongoing epithelial-to-mesenchymal transition, suggesting that the ER stress-induced intracellular pathological processes can also promote the acquisition of the mesenchymal phenotype. The role of chronic induction of the ER stress-induced UPR in ADF has been poorly studied with controversial results [22,23]. The recent interesting functional study of Consolato et al. demonstrated that missense mutations affecting α-Gal A folding promoted a gain-of-function effect due to ER stress leading to URP activation, suggesting that it could represent a novel pathogenic pathway in AFD in addition to the cellular damage due to lysosomal storage [24].

Female mutation carriers usually present milder phenotypes than comparable males, which might be explained by the skewed X inactivation, favoring the non-pathologically affected allele [25]. Regarding our index patient, laboratory data and clinical features give a mixed picture regarding disease patterns. The Asn5Asp variant is associated with almost all characteristic features described in female AFD patients, such as normal α-GLA activity and plasma lysoGb3 levels, nephrotic-range proteinuria, reduced renal function, left ventricular hypertrophy, and acroparesthesia [26]. This variant seems to have a role in the activation of the inflammatory cascade occurring through a further mechanism, ER stress, that should be considered in addition to the cellular damage due to lysosomal storage. Our hypothesis is strengthened by the evidence that, compared to a healthy control, the patient’s PBMCs exhibited higher expression levels of both TGF-β1 and pNFkB. This finding fits well with the phenotype of our patient, although, to date, the genotype–phenotype correlation in AFD patients has not been confirmed yet. The preliminary results of this in vitro study do not clarify the potential pathogenic role of the variant. Additionally, we have not conducted studies to verify whether the variant in the SP can cause alterations in protein folding, which in turn could lead to ER stress. However, the increase in GRP78 and certain markers of apoptosis, inflammation, and fibrosis suggest that the variant may activate the URP complex that switches its protective role to detrimental intracellular signals.

In conclusion, our findings suggest that the detrimental effect downstream of chronic UPR induction could represent a further candidate mechanism involved in the pathogenesis of tissue damage and that lysosomal storage is not the unique player in the organ failure observed in ADF disease, as already postulated in the literature recently.

## Data Availability

Data cannot be made publicly available because, in rare diseases, information about the diagnosis in combination with personal information may compromise the anonymity and confidentiality of the participants.
